# Scanning electron microscopy of the neuropathology of murine cerebral malaria

**DOI:** 10.1186/1475-2875-5-116

**Published:** 2006-11-24

**Authors:** Peter Lackner, Ronny Beer, Raimund Helbok, Gregor Broessner, Klaus Engelhardt, Christian Brenneis, Erich Schmutzhard, Kristian Pfaller

**Affiliations:** 1 Clinical Department of Neurology, Innsbruck Medical University, Innsbruck, Austria; 2 Division of Histology and Embryology, Innsbruck Medical University, Innsbruck, Austria

## Abstract

**Background:**

The mechanisms leading to death and functional impairments due to cerebral malaria (CM) are yet not fully understood. Most of the knowledge about the pathomechanisms of CM originates from studies in animal models. Though extensive histopathological studies of the murine brain during CM are existing, alterations have not been visualized by scanning electron microscopy (SEM) so far. The present study investigates the neuropathological features of murine CM by applying SEM.

**Methods:**

C57BL/6J mice were infected with *Plasmodium berghei ANKA *blood stages. When typical symptoms of CM developed perfused brains were processed for SEM or light microscopy, respectively.

**Results:**

Ultrastructural hallmarks were disruption of vessel walls, parenchymal haemorrhage, leukocyte sequestration to the endothelium, and diapedesis of macrophages and lymphocytes into the Virchow-Robin space. Villous appearance of observed lymphocytes were indicative of activated state. Cerebral oedema was evidenced by enlargement of perivascular spaces.

**Conclusion:**

The results of the present study corroborate the current understanding of CM pathophysiology, further support the prominent role of the local immune system in the neuropathology of CM and might expose new perspectives for further interventional studies.

## Background

Cerebral malaria (CM) is a major cause of mortality and morbidity in severe *Plasmodium falciparum *malaria. Frequently seen neurological dysfunctions are delirium, convulsions, coma and eventual death if the disease is not controlled. Post mortem analyses of brains of CM patients show adherence of parasitized red blood cells (pRBC) and inflammatory cells to the microvasculature of the brain, parenchymal microhaemorrhages and oedema. The pathophysiological mechanisms of CM are still discussed controversially. However, most researchers agree that two main factors contribute to the development of CM. On the one hand sequestration of blood cells (i.e. pRBC, leukocytes and thrombocytes) on activated endothelia causes obstruction of microvascular flow leading to local hypoxia [1,2]. On the other hand, excessively elevated cytokines in serum lead to activation of brain resident microglial cells which trigger local inflammatory processes [3-5]. Most of the knowledge about the pathophysiological mechanisms originates from studies in animal models (i.e. rodents) since the early stages of the disease are neuropathologically not addressable in humans. Hence the histopathology of murine CM has been studied in detail [6-8]. Ultrastructural analyses of different features of CM have been conducted. Transmission electron microscopy (TEM) provided direct evidence for pRBC sequestration and leukocyte attachment to the brain microvasculature [9]. Loosening of tight junctions contributing to brain oedema was confirmed by this method [10]. Haemorrhage attributable to disrupted brain vessels and enlargement of perivascular spaces has been observed in animals submitted to TEM analysis [11,12]. However, the above described features have not been visualized by scanning electron microscopy (SEM) so far.

Therefore, this study describes the pathological hallmarks of murine CM by means of SEM which offers the opportunity to analyse surfaces at high resolution and provides a three-dimensional view of the tissue structure.

## Methods

### Animals

Six C57BL/6J mice were infected intraperitoneally with 5*10^6 ^parasitized erythrocytes of a homologue donor, which had been infected with frozen polyclonal stocks of *Plasmodium berghei *ANKA [13]. Parasitaemia was monitored every other day by thin blood smears of tail blood stained with eosin methylene blue according to Wright (Sigma, St. Louis, MO, USA). When typical symptoms of CM (i. e. convulsions, paralysis of the limbs and coma) developed at day 6 post infection (p.i.), animals were terminally anesthetized with thiopental and transcardially perfused through the left ventricle (20 ml phosphate-buffered saline, pH 7.4 followed by 50 ml 3% glutaraldehyde in phosphate-buffered saline). The brains were removed carefully, post-fixed by immersion for 48 hours and then processed for SEM or light microscopy, respectively. Six non-infected animals were used as controls and processed exactly as animals with CM. All animal studies conformed to the Austrian guidelines for the care and use of laboratory animals and were approved by the Austrian Ministry for Education, Science and Culture.

### Light microscopy

Ten μm thick sections were cut on a cryotome and processed for routine staining (hematoxylin and eosin), in order to verify the histopathology in light microscopy.

Tissue for semi-thin sectioning was postfixed in 1% unbuffered aqueous osmium tetroxide, for one to two hours at 4°C and dehydrated in graded ethanol series. After dehydration, specimens were infiltrated with graded series of Epon epoxy resin. Resin was polymerized overnight at 60°C. Ribbons of semi-thin sections (0.5 μm) were made on a Reichert Ultracut S microtome (Leica Microsystems, Wetzlar, Germany) with a "Histo Jumbo Diamond" knife (Diatome, Biel, Switzerland) [14], and stained with toluidine blue.

### Field emission scanning electron microscopy

Fixed samples were sectioned coronally at 1 mm intervals using a mouse brain matrix (ASI Instruments Inc, Warren, USA). After a short wash in buffer the specimens were postfixed in 1% unbuffered aqueous osmium tetroxide, dehydrated in graded ethanol, critical point dried and attached to aluminium stubs. The specimens were coated in a Baltech MED020 Coating System with gold-palladium to a nominal depth of 10–12 nm and viewed in a Zeiss DSM982 Gemini Field Emission Electron Microscope operating at 5 kV. Maximal resolution at this voltage was estimated to 2 nm. Digital photos were taken at 1280 × 1024 resolution in TIFF format.

## Results

**Figure 1:**
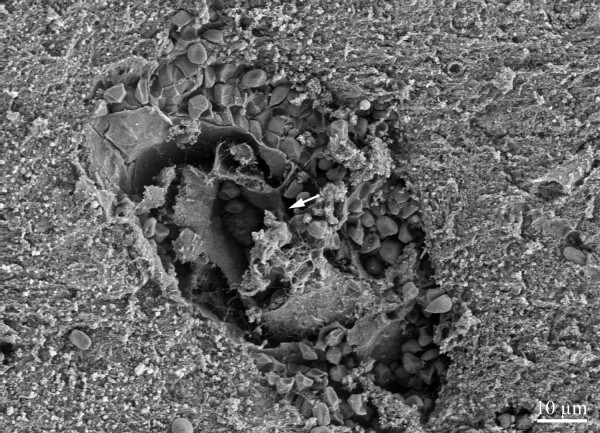
Intact erythrocytes surrounding a capillary in the cerebral cortex. Endothelial cell layer appears to have disintegrated (arrow).

**Figure 2:**
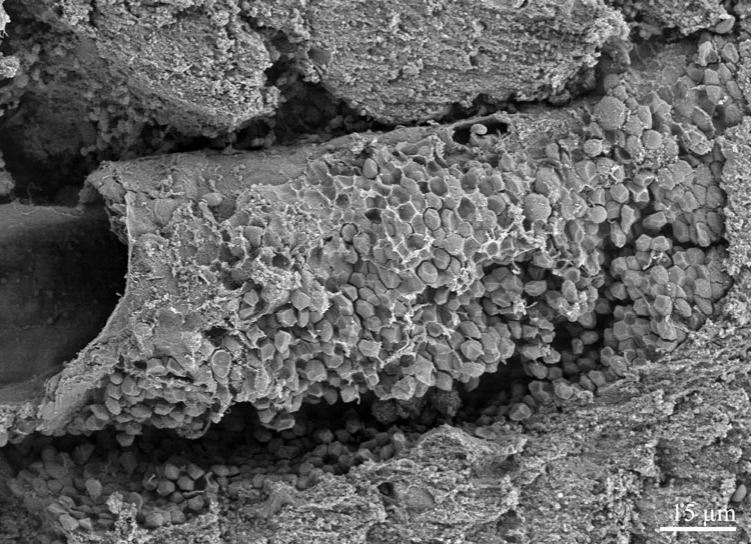
Parenchymal haemorrhage surrounding a brain arteriole. The vessel wall seems to be intact.

**Figure 3:**
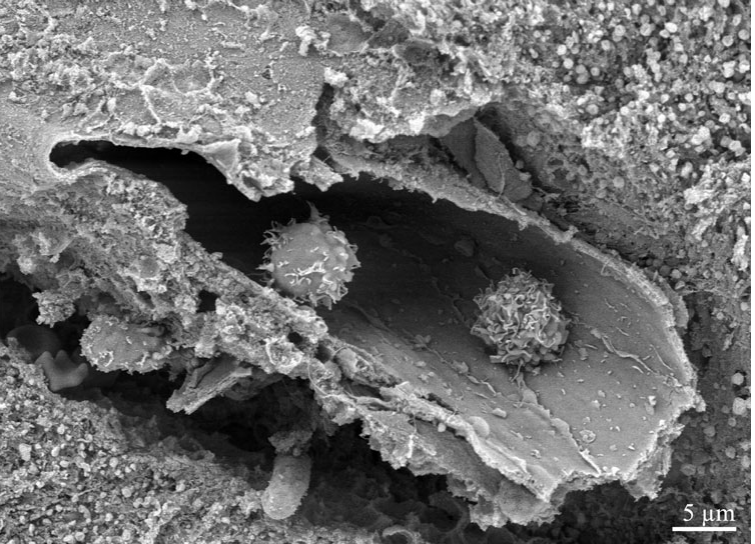
Two lymphocytes with villous surface (indicative of activated state [21]) sequestered in a brain vessel.

**Figure 4:**
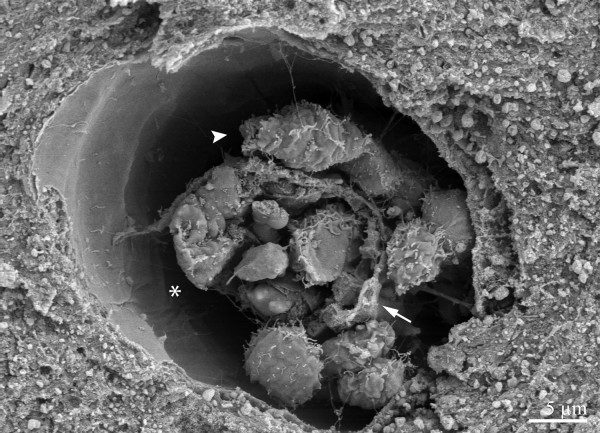
Capillary in cerebral cortex with sequestered leukocytes (arrow indicating vessel wall). Enlarged perivascular space (*) containing leukocytes attached to the vessel wall (arrowhead).

**Figure 5:**
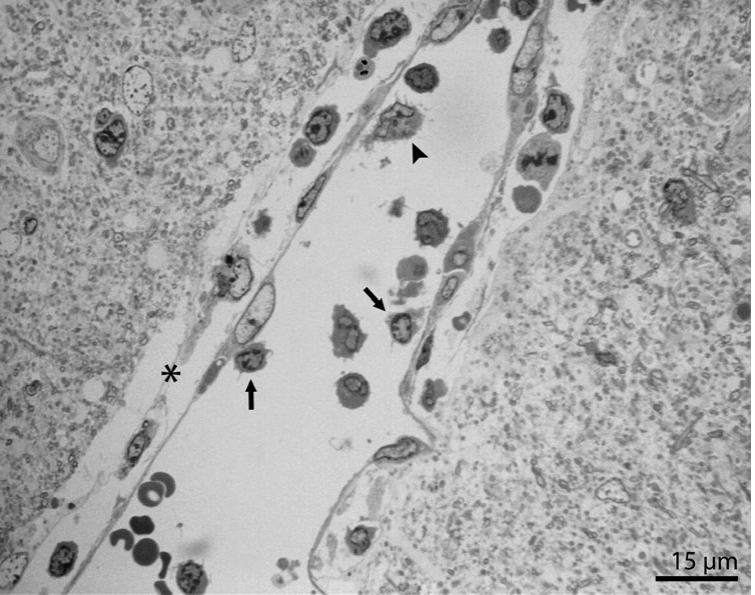
Semi-thin section of capillary in brainstem. Enlarged perivascular space (*) containing leukocytes in close vicinity to the vessel. Lymphocytes (arrows) and monocyte (arrowhead) sequestered to the endothelial wall.

**Figure 6:**
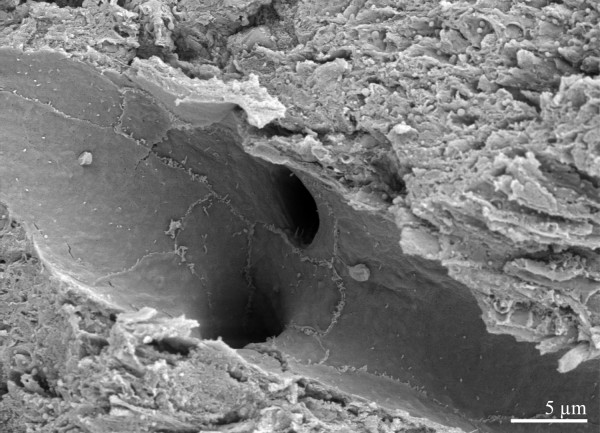
Capillary of an uninfected control animal. No sequestration of leukocytes or signs of perivascular inflammation and haemorrhage can be observed.

Six days p.i., infected animals developed signs and symptoms characteristic for CM, typically showing low levels of parasitaemia (5–15%). Gross examination of the brains revealed petechial bleedings on the brain surface, as well as generally swollen oedematous appearance. H&E staining of brains of animals with CM showed parenchymal microhaemorrhages and subarachnoid bleedings, disruption of vessel walls and cerebral oedema as indicated by enlargement of perivascular spaces and adherence of blood leukocytes to brain vessels as previously reported [15]. Consistent with these findings, SEM studies revealed disruption of vessel walls accompanied by haemorrhage (Figure 1). Some vessels surrounded by haemorrhages did not show evidence of vessel wall damage (Figure 2). Furthermore, sequestered leukocytes were a common feature (Figures 3, 4, 5). Analysis of semi-thin sections confirmed these leukocytes being predominantly monocytes and lymphocytes (Figure 5). Sequestered lymphocytes showed villous surface appearance (Figure 3). Enlarged perivascular spaces were indicative of brain oedema (Figures 4, 5). In addition, perivascular spaces frequently contained leukocytes (Figures 4, 5). In contrast, vessels of uninfected control animals showed neither sequestration of leukocytes nor signs of perivascular inflammation or haemorrhage, respectively (Figure 6).

## Discussion

A comprehensive morphological analysis of brain histopathology of murine CM by means of SEM is provided. To our knowledge, the present study is the first to apply SEM in investigating the histopathological features of murine CM. Prominent vascular pathology was present throughout the brain. Parenchymal haemorrhage seemed to be closely related to disrupted microvessels, which has been demonstrated previously [6,10].

Interestingly, ultrastructural evidence was found that in CM animals vessels without apparent wall injury may give rise to haemorrhage. This observation might be attributable to partial breakdown of the blood brain barrier, which is a common feature of murine CM [16-18]. Therefore, these findings support the hypothesis that not only thrombembolic obstruction and subsequent disintegration of microvessels but also extravasation through vessel walls with increased permeability leads to the frequently observed haemorrhages in CM. Since SEM can only inspect a small section of the whole vessel course, it cannot definitely be ruled out that tissue damage in close vicinity to the analysed regions could give rise to the observed haemorrhages.

In addition, SEM analysis yielded a high frequency of sequestered leukocytes (predominantly villous lymphocytes and monocytes) in brain microvasculature. In rodent CM leukocytes have been shown to be more abundantly sequestered than erythrocytes which is in contrast to human CM [19]. Sequestered lymphocytes are recognized to be causally related to the pathogenesis of murine CM. Moreover, the onset of neurological signs and symptoms is paralleled by the sequestration of CD8+ T-lymphocytes in the brain, and depletion of these cells, in contrast to neutrophil or macrophage depletion, confers protection against CM [20].

In the present study, most of the sequestered lymphocytes were of villous appearance. There is evidence that such lymphocyte surface morphology indicates activated state [21]. Furthermore, accumulation of activated T cells in the brains of CM mice has been shown by immunohistochemical analysis using the activation markers CD25 and CD54 [22]. Activated T-cells can induce local production of IFN-gamma and TNF-alpha and putatively NO, which leads to endothelial degeneration [23-25]. Thus, a critical issue in the pathogenesis of CM, namely the preferential recruitment of activated T cells in the cerebral microvasculature is confirmed by the current findings.

Another striking observation in the present study is enlargement of the perivascular spaces (also known as the Virchow-Robin space), which is commonly interpreted as a consequence of increased vascular permeability [6,12,26]. Interestingly, perivascular space frequently contained macrophages and lymphocytes abutting on the vascular endothelial sheet with their processes. Accumulation of inflammatory cells in Virchow Robin space occurs in various disease conditions. Perivascular inflammation is invariably found in experimental allergic encephalomyelitis, a well documented animal model for demyelinating diseases [27]. In a primate immunodeficiency model accumulation of macrophages ensheathing cerebral vessels has been recognized as a major contributor to the neuropathogenesis of AIDS [28].

There is consensus that perivascular cells are potential sensors of systemic inflammation [29]. These results taken together with the current findings suggest that during the early immune response to the parasite macrophages in the vicinity to the blood brain barrier are activated [5] and maintain the local inflammation leading to neuronal and glial dysfunction.

## Conclusion

In conclusion, SEM analysis of the murine brain in CM revealed a wide spectrum of pathological alterations and corroborates the data obtained from TEM and immunohistological analyses. The present morphological study further supports the prominent role of the local immune system in the neuropathology of murine CM. Ongoing studies applying SEM in CM as well as other infectious diseases of the CNS might promote a better understanding of the complex pathophysiological mechanisms leading to impaired neurological function.

## Authors' contributions

LP performed all animal experimentations, performed scanning electron microscopy and helped to draft the manuscript.

BR conducted light microscopy and helped to draft the manuscript.

HR helped to draft the manuscript.

BG performed image editing and helped to draft the manuscript.

EK helped to draft the manuscript.

BC helped to draft the manuscript.

SE helped to draft the manuscript.

PK performed scanning electron microscopy and helped to draft the manuscript.

## References

[B1] Grau GE, de Kossodo S (1994). Cerebral malaria: mediators, mechanical obstruction or more?. Parasitol Today.

[B2] Sanni LA, Rae C, Maitland A, Stocker R, Hunt NH (2001). Is ischemia involved in the pathogenesis of murine cerebral malaria?. Am J Pathol.

[B3] Medana IM, Hunt NH, Chan-Ling T (1997). Early activation of microglia in the pathogenesis of fatal murine cerebral malaria. Glia.

[B4] Jennings VM, Actor JK, Lal AA, Hunter RL (1997). Cytokine profile suggesting that murine cerebral malaria is an encephalitis. Infect Immun.

[B5] Pais TF, Chatterjee S (2005). Brain macrophage activation in murine cerebral malaria precedes accumulation of leukocytes and CD8+ T cell proliferation. J Neuroimmunol.

[B6] Jennings VM, Lal AA, Hunter RL (1998). Evidence for multiple pathologic and protective mechanisms of murine cerebral malaria. Infect Immun.

[B7] Medana IM, Chaudhri G, Chan-Ling T, Hunt NH (2001). Central nervous system in cerebral malaria: 'Innocent bystander' or active participant in the induction of immunopathology?. Immunol Cell Biol.

[B8] Carvalho LJ, Lenzi HL, Pelajo-Machado M, Oliveira DN, Daniel-Ribeiro CT, Ferreira-da-Cruz MF (2000). *Plasmodium berghei*: cerebral malaria in CBA mice is not clearly related to plasma TNF levels or intensity of histopathological changes. Exp Parasitol.

[B9] Hearn J, Rayment N, Landon DN, Katz DR, de Souza JB (2000). Immunopathology of cerebral malaria: morphological evidence of parasite sequestration in murine brain microvasculature. Infect Immun.

[B10] Ma N, Hunt NH, Madigan MC, Chan-Ling T (1996). Correlation between enhanced vascular permeability, up-regulation of cellular adhesion molecules and monocyte adhesion to the endothelium in the retina during the development of fatal murine cerebral malaria. Am J Pathol.

[B11] Rest JR, Wright DH (1979). Electron microscopy of cerebral malaria in golden hamsters (*Mesocricetus auratus*) infected with *Plasmodium berghei*. J Pathol.

[B12] Jacobs T, Plate T, Gaworski I, Fleischer B (2004). CTLA-4-dependent mechanisms prevent T cell induced-liver pathology during the erythrocyte stage of *Plasmodium berghei *malaria. Eur J Immunol.

[B13] Engwerda CR, Mynott TL, Sawhney S, de Souza JB, Bickle QD, Kaye PM (2002). Locally up-regulated lymphotoxin alpha, not systemic tumor necrosis factor alpha, is the principle mediator of murine cerebral malaria. J Exp Med.

[B14] Blumer MJ, Gahleitner P, Narzt T, Handl C, Ruthensteiner B (2002). Ribbons of semithin sections: an advanced method with a new type of diamond knife. J Neurosci Methods.

[B15] Lackner P, Beer R, Heussler V, Goebel G, Rudzki D, Helbok R, Tannich E, Schmutzhard E (2006). Behavioural and histopathological alterations in mice with cerebral malaria. Neuropathol Appl Neurobiol.

[B16] Adams S, Brown H, Turner G (2002). Breaking down the blood-brain barrier: signaling a path to cerebral malaria?. Trends Parasitol.

[B17] Ma N, Madigan MC, Chan-Ling T, Hunt NH (1997). Compromised blood-nerve barrier, astrogliosis, and myelin disruption in optic nerves during fatal murine cerebral malaria. Glia.

[B18] Chang-Ling T, Neill AL, Hunt NH (1992). Early microvascular changes in murine cerebral malaria detected in retinal wholemounts. Am J Pathol.

[B19] de Souza JB, Riley EM (2002). Cerebral malaria: the contribution of studies in animal models to our understanding of immunopathogenesis. Microbes Infect.

[B20] Belnoue E, Kayibanda M, Vigario AM, Deschemin JC, Van Rooijen N, Viguier M, Snounou G, Renia L (2002). On the pathogenic role of brain-sequestered alphabeta CD8+ T cells in experimental cerebral malaria. J Immunol.

[B21] van Ewijk W, Brons NH, Rozing J (1975). Scanning electron microscopy of homing and recirculating lymphocyte populations. Cell Immunol.

[B22] Haque A, Echchannaoui H, Seguin R, Schwartzman J, Kasper LH, Haque S (2001). Cerebral malaria in mice: interleukin-2 treatment induces accumulation of gammadelta T cells in the brain and alters resistant mice to susceptible-like phenotype. Am J Pathol.

[B23] Christmas SE, Meager A (1990). Production of interferon-gamma and tumour necrosis factor-alpha by human T-cell clones expressing different forms of the gamma delta receptor. Immunology.

[B24] Ferrick DA, Schrenzel MD, Mulvania T, Hsieh B, Ferlin WG, Lepper H (1995). Differential production of interferon-gamma and interleukin-4 in response to Th1- and Th2-stimulating pathogens by gamma delta T cells in vivo. Nature.

[B25] Jones-Carson J, Vazquez-Torres A, van der Heyde HC, Warner T, Wagner RD, Balish E (1995). Gamma delta T cell-induced nitric oxide production enhances resistance to mucosal candidiasis. Nat Med.

[B26] Medana IM, Chan-Ling T, Hunt NH (1996). Redistribution and degeneration of retinal astrocytes in experimental murine cerebral malaria: relationship to disruption of the blood-retinal barrier. Glia.

[B27] Matsumoto Y, Fujiwara M (1987). The immunopathology of adoptively transferred experimental allergic encephalomyelitis (EAE) in Lewis rats. Part 1. Immunohistochemical examination of developing lesions of EAE. J Neurol Sci.

[B28] Williams KC, Corey S, Westmoreland SV, Pauley D, Knight H, deBakker C, Alvarez X, Lackner AA (2001). Perivascular macrophages are the primary cell type productively infected by simian immunodeficiency virus in the brains of macaques: implications for the neuropathogenesis of AIDS. J Exp Med.

[B29] Williams K, Alvarez X, Lackner AA (2001). Central nervous system perivascular cells are immunoregulatory cells that connect the CNS with the peripheral immune system. Glia.

